# Human gastric cancer progression and stabilization of ATG2B through RNF5 binding facilitated by autophagy-associated CircDHX8

**DOI:** 10.1038/s41419-024-06782-8

**Published:** 2024-06-12

**Authors:** Guanxin Wei, Xiang Chen, Tuo Ruan, Xianxiong Ma, Xiuxian Zhu, Wenhao Wen, Danzeng He, Kaixiong Tao, Chuanqing Wu

**Affiliations:** Department of Gastrointestinal Surgery, Union Hospital, Tongji Medical College, Huazhong University of Science and Technology, Wuhan, 430022 China

**Keywords:** Targeted therapies, Chaperone-mediated autophagy

## Abstract

The role of circDHX8 in the interplay between autophagy and gastric cancer (GC) progression remains unclear. In this study, we investigated the mechanism underlying the role of hsa_circ_003899 (circDHX8) in the malignancy of GC. Differential expression of circRNAs between GC and normal tissues was determined using circle-seq and microarray datasets (GSE83521). These circRNAs were validated using qPCR and Sanger sequencing. The function of circDHX8 was investigated through interference with circDHX8 expression experiments using in vitro and in vivo functional assays. Western blotting, immunofluorescence, and transmission electron microscopy were used to establish whether circDHX8 promoted autophagy in GC cells. To elucidate the mechanism underlying the circDHX8-mediated regulation of autophagy, we performed bioinformatics analysis, RNA pull-down, mass spectrometry (MS), RNA immunoprecipitation (RIP), and other western Blot related experiments. Hsa_circ_0003899 (circDHX8) was identified as upregulated and shown to enhance the malignant progression in GC cells by promoting cellular autophagy. Mechanistically, circDHX8 increased ATG2B protein levels by preventing ubiquitin-mediated degradation, thereby facilitating cell proliferation and invasion in GC. Additionally, circDHX8 directly interacts with the E3 ubiquitin-protein ligase RNF5, inhibiting the RNF5-mediated degradation of ATG2B. Concurrently, ATG2B, an acetylated protein, is subjected to SIRT1-mediated deacetylation, enhancing its binding to RNF5. Consequently, we established a novel mechanism for the role of circDHX8 in the malignant progression of GC.

## Introduction

Gastric cancer (GC) is the fifth most prevalent caecause of few apparent symptoms in the early stagesncer globally and the third most fatal among all cancer types [1, 2]. Because of few apparent symptoms in the early stages, GC is typically detected at advanced stages. Advanced GC increases susceptibility to distant metastasis and postoperative recurrence, leading to a poor prognosis [1]. Research on the pathogenesis and development of GC has revealed that it represents highly heterogeneous diseases with abnormally mutated genes and signaling pathways [3]. Therefore, elucidating the specific mechanisms underlying GC occurrence and development is important.

As a highly conserved and ancient catabolic process, autophagy sequesters cytoplasmic proteins or organelles into vesicles and fuses them with lysosomes to form autophagy-lysosomes and initiate the degradation process of the contents of the package. This metabolism of the cell facilitates the recycling and renewal of some organelles [4]. Since Christian de Duve first proposed autophagy in 1963, it has been identified as associated with multitudinous diseases, particularly in cancer. In GC, autophagy activation promotes cancer progression and induces ubiquitination [5–8], which has long been recognized as a key factor in proteasomal degradation of tagged proteins and is also involved in selective autophagy. Expanding research on autophagy has demonstrated that ubiquitination could affect autophagy and induce cancer progression. For example, SPOP, the E3 ubiquitin ligase substrate-binding adapter, could suppress p62-dependent autophagy in prostate cancer (PCa) [9]; therefore, PCa-associated SPOP mutants could promote p62-dependent autophagy. E3 ubiquitin ligase CUL3 interacts with BECN1 to mediate its ubiquitination and degradation, reducing its protein expression level to inhibit cell autophagy and promoting tumor progression [10].

Circular RNAs (circRNAs), lacking a 5′ terminal cap and 3′ terminal poly(A) tail, are covalently bonded to form a circular structure [11, 12]. Evidence suggests that aberrant expression of circRNAs occurs in almost all types of cancer, either in a pro- or anti-tumor role, and circRNAs regulate the tumor immune microenvironment and metabolic reprogramming, influence the proliferation, invasion, migration and apoptosis of cancer cells, and affect the drug resistance of tumors [13, 14]. For instance, circRNA-SORE promotes sorafenib resistance in hepatocellular carcinoma [15], circRNA-CREIT inhibits doxorubicin resistance in breast cancer [16], and circNDUFB2 inhibits cancer progression in lung cancer [17]. In GC, CircPDIA4 was found to contribute to GC progression through a dual tumor-promoting mechanism [18]. As a circRNA with coding potential, CircMTHFD2L (also known as hsa_circ_0069982) encodes CM-248aa to restrain GC progression [19]. Furthermore, accumulating evidence suggests that circRNAs influence cancer progression by modulating autophagy [20–23]. However, the biological function and mechanism of circRNA modulation of autophagy remain unclear.

We aimed to elucidate the role of hsa_circ_0003899 (circDHX8) in gastric cancer and its specific mechanism. We combined cell research and clinical data to fully demonstrate the importance of circDHX8 in the occurrence and development of GC and provide new targets for precision treatment of GC.

## Materials and methods

### Patients and tissues

We studied human GC tissue samples from patients with GC undergoing tumor resection at Union Hospital of Tongji Medical School, Huazhong University of Science and Technology. The postoperative pathological diagnosis results were all confirmed as gastric adenocarcinoma. In accordance with the Declaration of Helsinki, this research was approved by the Ethics Committee of Union Hospital. All tissue donors provided written informed consent.

### Cell line origin and culture

All cell lines used were derived from Cobioer Biosciences (Nan Jing, China). All the cells were cultured in a humidified incubator at 37°C under 5% CO_2_. DMEM (Gibco, Carlsbad, CA, USA) was utilized for culturing GES-1 cells, and GC cell lines were cultured in RPMI-1640 (Gibco). Fetal bovine serum (10%; Gibco) was added to all media.

### Gene expression data

Gene expression data (GSE83521) were downloaded from the Gene Expression Omnibus database (http://www.ncbi.nlm.nih.gov/geo).Proteogenomics of Gastric Cancer - Proteome data were obtained from Clinical Proteomic Tumor Analysis Consortium (CPTAC, https://pdc.cancer.gov/pdc/PDC000214).

### Transfections and construction of SIRT1-H363Y

ATG2B-siRNA (ATG2B-KD), RNF5-siRNA (RNF5-KD), and a negative control were obtained from GeneChem (Shanghai, China). The ATG2B, RNF5 full-length sequence, and its truncations were composited by TSINGKE (Wuhan, China) and then inserted into the pcDNA3.1 (RiboBio) to establish overexpression plasmids, which were utilized for overexpression. The circDHX8 full-length sequence was composited by TSINGKE, cloned, and inserted into the pLCDHciR lentiviral vector (GeneChem). Two shRNAs for circDHX8 (sh-circDHX8-1 and sh-circDHX8-2) were transfected into GV298 (GeneChem) to knock down circDHX8 expression. Lipofectamine 3000 transfection reagent (Invitrogen, Carlsbad, CA, USA) promoted all shRNAs and overexpression plasmids transfection. SIRT1-H363Y was constructed using the Hieff Mut™ Site-Directed Mutagenesis Kit (Yi Sheng Biotechnology, Shanghai, China).

### Quantitative real-time PCR

According to the manufacturer’s instructions, RNAkeyTM Reagent (Seven, Beijing, China) was utilized to extract total RNA from cells or tissues. HiScript III RT SuperMix (Vazyme, Nanjing, China) was then used to reverse-transcribe total RNA to cDNA.

2×SYBR Green qPCR MasterMix II (Seven, Beijing, China) was used to run qPCR with an ABI StepOne Plus system (Thermo Fisher Scientific, Waltham, MA, USA). We used GAPDH for normalizing mRNA and circRNA levels, and the relative expression levels of each mRNA were calculated using the 2^−ΔΔCT^ method. Each experiment was conducted in triplicate.

### Immunofluorescence and fluorescence in situ hybridization

Immunofluorescence assays were performed as previously described [23] to detect the intracellular localization of ATG2B and RNF5 in MKN45 and AGS cells. Fluorescence in situ hybridization assays were performed using the fluorescence in situ hybridization kit for RNA (R0306S, Beyotime, Shanghai, China) according to the manufacturer’s instructions. RiboBio Technology Co. synthesized the biotin-labeled probes targeting circDHX8. A confocal laser scanning microscope (LSM 780 with Airyscan; Carl Zeiss, Oberkochen, Germany) was used for observation and to take pictures.

### Western blot analysis

Using RIPA lysis buffer (P0013B, Beyotime, Shanghai, China) to extract total proteins from cells, total proteins were separated by 10% Tris-glycine gels electrophoresis and then transferred onto a polyvinylidene fluoride membrane (MilliporeSigma, Burlington, MA, USA). Next, 5% skim milk was enclosed at room temperature for 1 h before being incubated overnight with the following primary antibodies: anti-SQSTM1/p62 antibody (ab109012, Abcam, Cambridge, UK), anti-LC3B antibody (ab192890, Abcam), anti-GAPDH antibody (60004-1-Ig, Proteintech, Beijing, China), anti-Myc tag antibody (ab32, Abcam), anti-GADD34/PPP1R15A antibody (ab9869, Abcam), anti-CDKN1A/P21 antibody (ab109520), Abcam, anti-c-FOS antibody (ab208942, Abcam), anti-PRKCQ antibody (ab230972, Abcam), anti-RAB7 antibody (ab137029, Abcam), anti-ATG2B antibody (ab189934, Abcam), anti-Ubiquitin antibody (ab134953, Abcam), anti-RNF5 antibody (ab308066, Abcam), anti-SIRT1 antibody (ab110304, Abcam), and anti-Actin antibody (Cat# AA128, Beyotime, Shanghai, China). After washing thrice with TBST, goat anti-Rabbit IgG-HRP antibody (Abcam) was used as a secondary antibody. PBST was used to wash the film twice and PBS was used to wash the film for 10 min, and visualization was finally performed with an enhanced chemiluminescence system (Millipore).

### Colony formation assays

Single layer cultured GC cells with logarithmic growth stage were taken, digested with 0.25% trypsin, and blown into single cells, and the cells were suspended in RPMI1640 culture medium with 10% fetal bovine serum. Digested GC cells (2000/well) were then inoculated into 6-well plates containing 2 mL of culture medium and incubated at 37°C, 5% CO2, and saturated humidity for 2 to 3 weeks. The culture was terminated when visible clones appeared in the petri dish using methanol 5 mL and fixing for 15 min. An appropriate amount of 0.1% crystal violet was added for 10–30 min, the plate was turned upside down, and a transparent film with a mesh was superimposed to count the clones either directly with the naked eye or under a microscope (at low power) with more than 50 cells. Finally, the clone formation rate was calculated. The formula for the clone formation rate was as follows: number of clones/number of inoculated cells ×100%.

### CCK8

Following the manufacturer’s protocol (Dojondo Laboratories, Kumamoto, Japan), cell suspension (100 μL/well) was inoculated in a 96-well plate. The plates were pre-cultured in an incubator (37 °C, 5% CO_2_). Next, 10 μL CCK solution was added to each hole, and the plates were incubated for 1 to 4 h. The absorbance at 450 nm was measured by an enzyme-labeling instrument (Bio-Rad Laboratories, Hercules, CA, USA).

### Transwell assay

Single layer cultured GC cells with logarithmic growth stage were taken, digested with 0.25% trypsin, and blown into single cells. The cells were then suspended in RPMI1640 culture medium with 10% fetal bovine serum. Thereafter, the cells were resuspended in serum-free medium, and the cell density was adjusted to 2.5 × 10^5^/mL after counting. Next, 500 μL of conditioned medium containing 20% FBS was added to the lower chamber. A transwell chamber (Corning Life Sciences, Corning, NY, USA) was then placed inside a 24-well plate. Each well in the upper chamber had 100 μL of cell suspension added. After incubating at 37 °C for 20–24 h, the transwell chamber was removed, washed twice with PBS, and fixed with 5% glutaraldehyde at 4 °C. Next, 0.1% crystal violet was added for staining. Subsequently, the cells were observed under a microscope, and images were captured and analyzed using ImageJ software (National Institutes of Health, Bethesda, MD, USA).

### Cell cycle: propidium iodide staining

Cell cycle distribution was determined by flow cytometry. Growth stage GC cells were collected, 500 μL PBS was added to gently blow the cell clusters into cell suspensions, and 2 mL cold 95% ethanol at 20 °C was added drop by drop in the vortex state, mixed and fixed for 30 min. Next, 5 mL of PBS was added and centrifuged at 1500 rpm for 5 min to remove the supernatant. Cells were resuspended by adding 5 mL of PBS and centrifuged at 1500 rpm for 5 min to remove the supernatant; 800 uL propidium iodide staining solution was added, gently blowing the cell group with guns, blending, and dyeing for 30 min at room temperature away from light. Red fluorescence was detected using flow cytometry (BD Biosciences, Franklin Lakes, NJ, USA) at an excitation wavelength of 488 nm (PE channel).

### TUNEL staining assays

The MKN45/AGS cells were seeded in 24-well plates, according to the manufacturer’s instructions (One-step TUNEL In Situ Apoptosis Kit, Elabscience, Wuhan, China). The results were captured using a fluorescence microscope (Leica Microsystems, Wetzlar, Germany).

### RNA pull‑down and mass spectrometry

The specific procedure was consistent with that described in a previous paper [24].

### RNA immunoprecipitation

RNA immunoprecipitation (RIP) assays were performed according to the manufacturer’s instructions (Millipore, MA, USA). Briefly, GC cells were lysed by RIP lysis buffer, and were incubated with protein A/G agarose beads and RNF5 antibody or anti-AGO2 antibody (Millipore) overnight at 4°C. DNA electrophoresis was performed to verify the reverse transcription products of the co-precipitated RNA.

### Tumor Xenograft Assay

Our research obtained ethical approval from the Formal Review of Experimental Animal Ethics committee at Huazhong University of Science and Technology. BALB/c nude mice (male, 4 weeks) from Huafukang (Beijing, China) were used for the in vivo xenograft assays. All nude mice were randomly divided into two groups (sh-NC vs. sh-circDHX8-1), and GC cells (5 × 10^6^) were injected into the groins of the randomly grouped nude mice. The tumor volume was recorded every 5 days. After 25 days, the mice were euthanized, and the tumors were weighed and imaged.

### Transmission electron microscopy

GC cells were seeded and cultured in 6-well plates for different treatments. After glutaraldehyde osmic acid fixation, gradient ethanol and acetone dehydration, infiltration, embedding, high-temperature polymerization, cutting, semi-thin sectioning, staining, positioning, ultrathin sectioning, and uranyl acetate and lead citrate staining, cell samples were cut into ultrathin sections and subjected to transmission electron microscopy (transmission electron microscopy; Hitachi HT7700; Hitachi, Tokyo, Japan). Changes in the autophagosomes in the cells were observed and imaged.

### Statistical analysis

All statistical analyses and plots were conducted using GraphPad Prism 9 (GraphPad Software, La Jolla, CA, USA) and Excel (Microsoft). Data are presented as the mean ± SD, and Student’s *t*-tests were used to compare differences between two date groups. ANOVA tests were used for more than two date groups, and a log-rank test was implemented for survival analysis. *P* < 0.05 was considered statistically significant.

## Results

### Identification of hsa_circ_0003899 and its abnormally high expression in GC

**Fig. 1:**
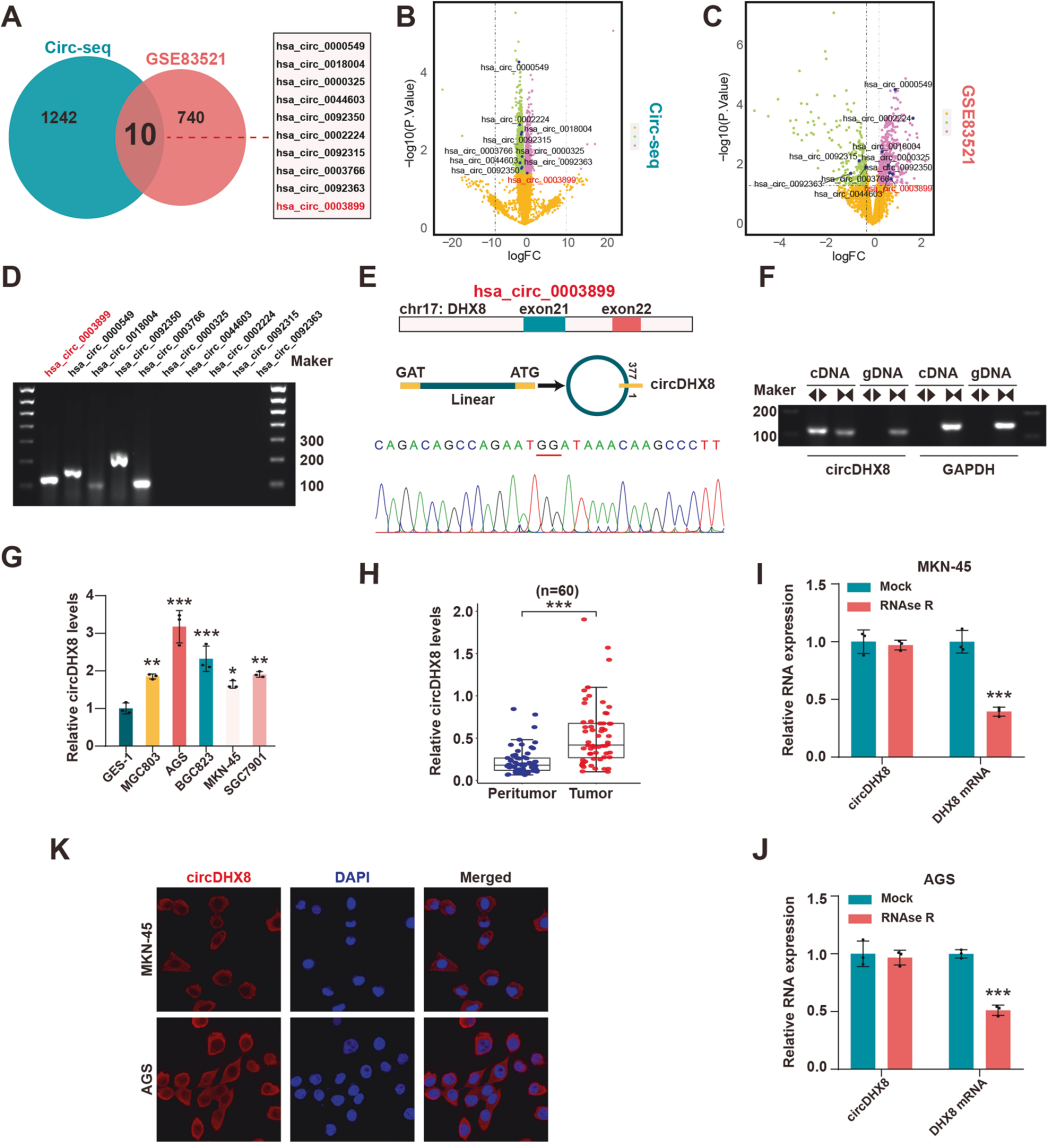
CircDHX8 is upregulated in gastric cancer (GC) tissues and cell lines and is localized in cell cytoplasm. **A**–**C** Microarray analysis of differentially expressed circRNAs (DECs) of GC and peritumor tissues from circ-seq and the GSE83521 dataset. **D** Results of DNA electrophoresis with the PCR products of the 10 DECs. **E**–**F** Sanger sequencing and DNA electrophoresis results with the PCR products of hsa_circ_0003899. **G** qPCR analysis of circDHX8 in GC cells and GES-1 cells. **H** qPCR analysis of circDHX8 in GC and peritumor tissues (*n* = 60). **I**,**J** qPCR analysis of circDHX8 and DHX8 mRNA in response to RNase R treatment. **K** RNA-FISH staining showed the cytoplasmic localization of circDHX8. *P < 0.05, **P < 0.01, and ***P < 0.001.

Given the crucial role of circRNAs in GC tumorigenesis, we utilized circRNA sequencing in GC and normal tissue samples (*n* = 60) and microarray datasets (GSE83521) to analyze differentially expressed circRNAs. Of these, 10 differentially expressed circRNAs were identified as candidates for further investigation (Fig. 1A–C), wherein we utilized divergent primers for cDNA to validate five circRNAs by DNA electrophoresis (Fig. 1D). We subsequently performed Sanger sequencing of the PCR products of these circRNAs and confirmed that three circRNAs (hsa_circ_0003899, hsa_circ_0018004, and hsa_circ_0003766) were consistent with sequences in the circBase database. Additionally, DNA electrophoresis confirmed the circRNAs circular structures (Fig. 1E, F; Fig. S1A–D). We further assessed the expression of these three circRNAs in normal gastric mucosa epithelial cells (GES-1), five GC cell lines, peritumor tissues, and GC tissue samples using qPCR. The results revealed hsa_circ_0003899 was significantly upregulated in GC tissues and cell lines (Fig. 1G, H; Fig. S1E–H), leading us to focus on hsa_circ_0003899, termed circDHX8, for subsequent studies. AGS and MKN45 GC cell lines were selected to facilitate interference with circDHX8 expression. We further confirmed its circular RNA structure after RNase R exonuclease digestion (Fig. 1I–J), and fluorescence in situ hybridization assays demonstrated its cytoplasmic localization (Fig. 1K). These findings indicate that circDHX8 is abnormally upregulated in GC and localizes in the cytoplasm.

### CircDHX8 generated tumor-promoting effects in GC cell lines

**Fig. 2:**
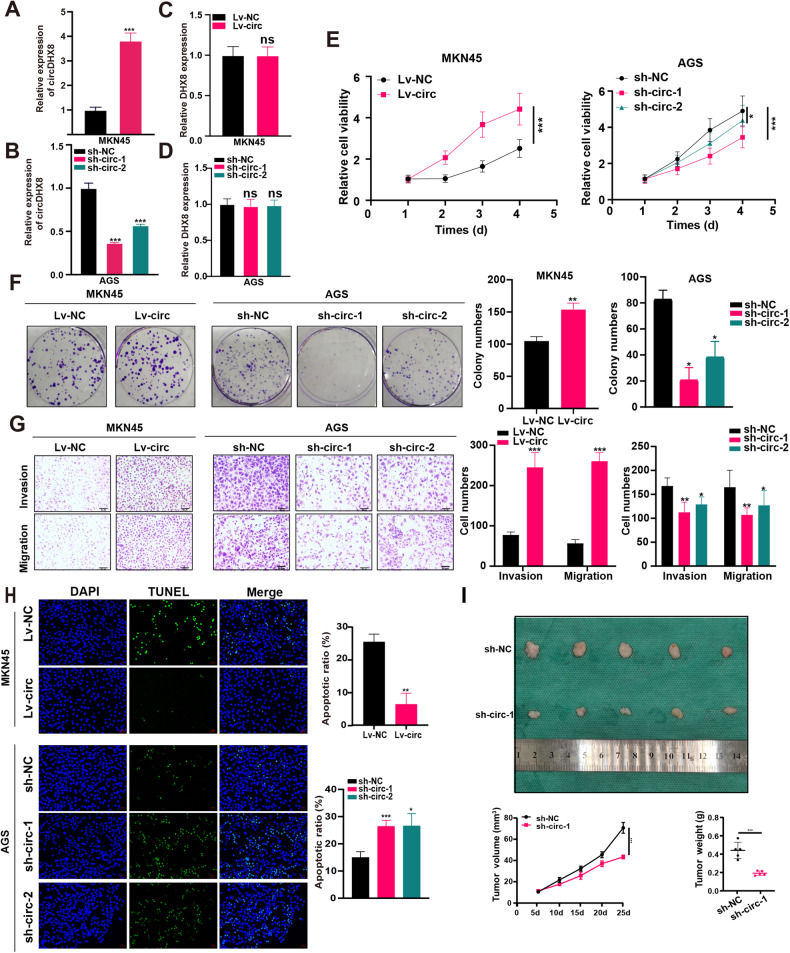
CircDHX8 promotes gastric cancer proliferation, migration, and invasion in vitro and in vivo*.* **A**,**B** qPCR analysis of circDHX8. **C**,**D** qPCR analysis of DHX8 expression. **E** Cell counting kit-8 assay analysis of cell proliferation in MKN45 and AGS cells after circDHX8 overexpression or knockdown. **F** Colony-forming capacity of MKN45 and AGS cells after circDHX8 overexpression or knockdown. **G** Transwell assays for migration and invasion in MKN45 and AGS cells. **H** Cell apoptosis was analyzed by TUNEL assays. **I** Subcutaneous xenograft of MKN45 cells after circDHX8 knockdown. *P < 0.05, **P < 0.01 and ***P < 0.001.

Given the elevated expression of circDHX8 in GC, we explored its specific role in GC tumorigenicity. We transfected circDHX8 plasmids or their corresponding shRNAs into two GC cell lines, MKN45 and AGS. qPCR was used to verify the successful modulation of circDHX8 expression in these cell lines post-transfection (Fig. 2A, B). To show that circDHX8, rather than the linear transcript DHX8, functions in GC progression, we determined the relative DHX8 expression after circDHX8 overexpression or knockdown. As shown in Fig. 2C, D, circDHX8 had no influence on linear transcript DHX8 expression.

Overexpression of circDHX8 markedly increased the proliferation, invasion, and migration ability of GC cells. Conversely, circDHX8 knockdown remarkably reduced these effects (Fig. 2E–G and Fig. S2A–C). Furthermore, circDHX8 overexpression substantially inhibited GC cell apoptosis, whereas its knockdown had the opposite effect (Fig. 2H). Stable circDHX8 knockdown in GC cells was achieved in vivo, and the results showed that circDHX8 knockdown markedly inhibited xenograft tumor growth (Fig. 2I).

### Biological functions of circDHX8 played in GC cells depending of autophagy induction

**Fig. 3:**
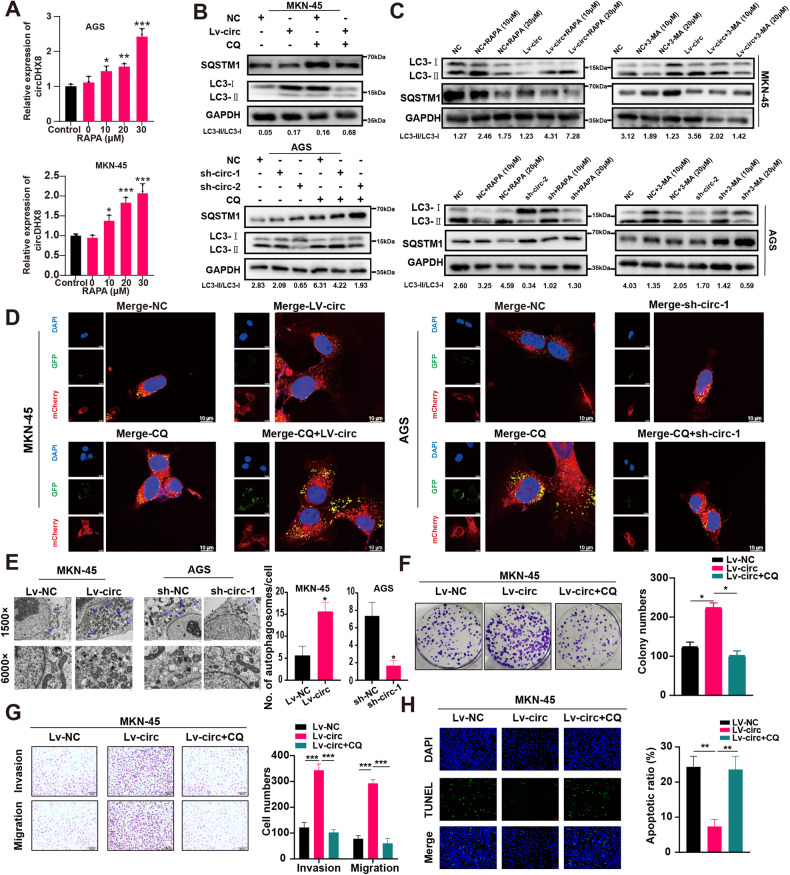
circDHX8 plays a biological role in gastric cancer cells via autophagy. **A** qPCR analysis of circDHX8 in MKN45 and AGS cells after RAPA treatment. **B**,**C** Western blotting analysis of the protein levels of LC3II/I and SQSTM1 in MKN45 and AGS cells. **D** Immunofluorescence staining of autophagosomes and autolysosomes. **E** Electron microscopy was used to observe autophagosomes. **F** Colony-forming capacity of MKN45. **G** Transwell assays for migration and invasion in MKN45 cells. **H** Cell apoptosis was analyzed by TUNEL assays. *P < 0.05, **P < 0.01 and ***P < 0.001.

Autophagy is essential in GC progression [5, 25, 26], and the autophagy activator rapamycin enhances circDHX8 expression. Therefore, we investigated whether circDHX8 induces autophagy in GC cell lines. The results of qPCR revealed a dose-dependent increase in circDHX8 expression in MKN45 and AGS cells upon treatment with rapamycin, an autophagy activator (Fig. 3A). Autophagy induction was validated by the ratio of LC3-II to LC3-I and other autophagic biomarkers, such as SQSTM1/P62, which serve as a link between polyubiquitinated cargo and autophagosomes [27].

We used chloroquine (CQ), a lysosomal inhibitor that impedes the fusion of autophagosomes with lysosomes and blocks lysosomal degradation, to rigorously confirm circDHX8’s role in autophagy induction. The results indicated that circDHX8 significantly augmented the LC3-II/LC3-I ratio in GC cell lines, with P62 levels being inversely related to LC3-II/LC3-I. CQ treatment led to increased accumulation of P62 and LC3II (Fig. 3B). To confirm these findings, GC cells were treated with rapamycin and 3-methyladenine, an autophagy inhibitor that blocks autophagosome formation. circDHX8 overexpression or knockdown upregulated or downregulated autophagic activity in MKN45 and AGS cells, as evidenced by the LC3-II/LC3-I ratio and P62 levels (Fig. 3C). Visualization of autophagosomes by confocal microscopy using mCherry and green fluorescence protein-tagged LC3 showed increased LC3 dots in MKN45 cells overexpressing circDHX8 and decreased dots in AGS cells following circDHX8 knockdown (Fig. 3D). Transmission electron microscopy analysis aligned with autophagy flux observations confirmed that circDHX8 substantially increased the number of autophagic vacuoles in AGS and MKN45 cells (Fig. 3E). Overall, our results suggest that circDHX8 could promote autophagy in GC cells. Subsequent experiments revealed that inhibiting autophagy using CQ significantly counteracted the impacts of circDHX8 on colony formation, invasion, migration, and apoptosis in MKN45 cells (Fig. 3F–H).

### CircDHX8 upregulates ATG2B protein levels by inhibiting its ubiquitination degradation to promote cell proliferation and invasion in GC

**Fig. 4:**
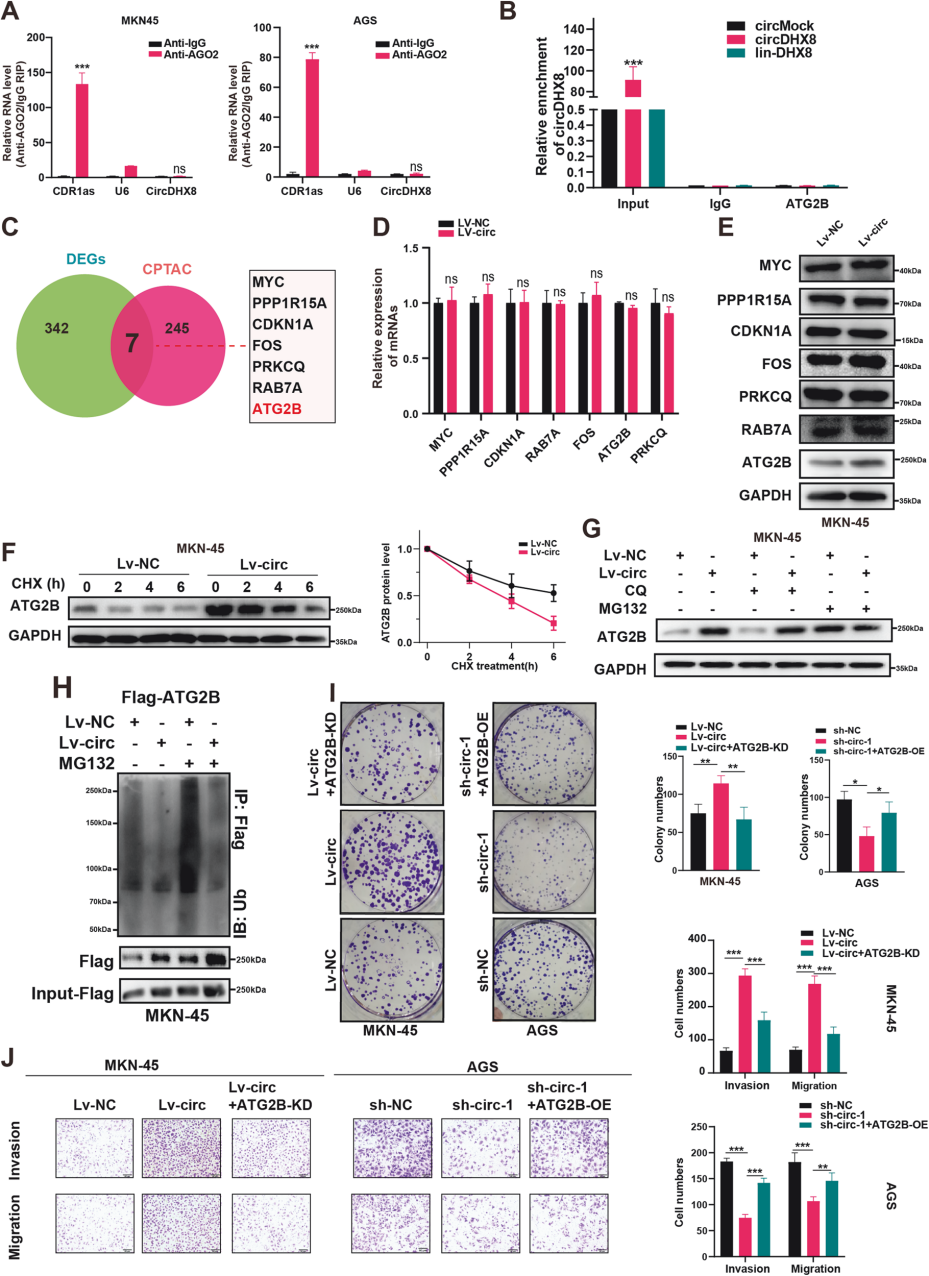
circDHX8 promotes autophagy by inhibiting ATG2B ubiquitination in gastric cancer. **A** qPCR analysis of circDHX8 with AGO2-RIP. **B** RIP analysis of the relative interplay between circDHX8 and ATG2B. **C** Overlapping analysis of differentially expressed genes (DEGs) between the circDHX8-OE and control groups with DEGs from CPTAC. **D** qPCR analysis of MYC, PPP1R15A, CDKN1A, RAB7A, FOS, ATG2B, and PRKCQ after circDHX8 overexpression. **E** Western blotting analysis of the protein levels of MYC, PPP1R15A, CDKN1A, RAB7A, FOS, ATG2B, and PRKCQ after circDHX8 overexpression. **F** Protein stability of ATG2B was assessed by western blotting. **G** The effect of circDHX8 and chloroquine on ATG2B protein levels was analyzed by western blotting. **H** Western blotting assays showed that circDHX8 could suppress the ubiquitination of ATG2B. **I** Colony-forming capacity of MKN45 and AGS cells. **J** Transwell assays for migration and invasion in MKN45 and AGS cells. *P < 0.05, **P < 0.01 and ***P < 0.001.

Subsequently, we performed a RIP assay with an Ago2 antibody to investigate whether circDHX8 plays a function as a miRNA sponge. However, endogenous circDHX8 was not significantly enriched by Ago2 antibodies (Fig. 4A). To identify potential targets related to the effect of circDHX8 on GC cells, we analyzed the differentially expressed genes in AGS cells transfected with shRNAs (sh-circ#1) and a negative control (sh-NC) that overlapped with the CPTAC database (PDC000214) between GC and normal tissues. We identified seven differentially expressed molecules and assessed the impact of circDHX8 on their expression in MKN45 using western blotting and RT-qPCR. However, we noticed that circDHX8 had no significant effect on the mRNA level of these molecules but increased the protein level of ATG2B (Fig. 4C–E). This suggests that transcriptional regulation may not be involved in the reduction in ATG2B protein levels. This finding prompted us to speculate whether circDHX8 affects the ATG2B degradation process. However, circDHX8 did not directly bind to ATG2B (Fig. 4B; Fig. S3A). Cycloheximide chase assays revealed that circDHX8 overexpression increased the protein stability of ATG2B. Specifically, after the inhibition of proteasome activity by MG132 in MKN45 cells, circDHX8 overexpression did not further increase ATG2B protein levels relative to controls without MG132 or CQ treatment. Nevertheless, circDHX8 knockdown in AGS cells showed the opposite results (Fig. 4F, G; Fig. S3B, C). Next, we investigated the impact of circDHX8 on ATG2B ubiquitination and found that circDHX8 could suppress the ubiquitination of ATG2B (Fig. 4H; Fig. S3D). These results suggested that circDHX8 stabilizes ATG2B by inhibiting its ubiquitination and degradation. Subsequently, cell function experiments confirmed that circDHX8 plays a role in GC cells via ATG2B (Fig. 4I-J; Fig. S3E–H).

### CircDHX8 directly binds with E3 Ubiquitin-Protein Ligase RNF5 to inhibit the RNF5-mediated ATG2B ubiquitin degradation pathway

**Fig. 5:**
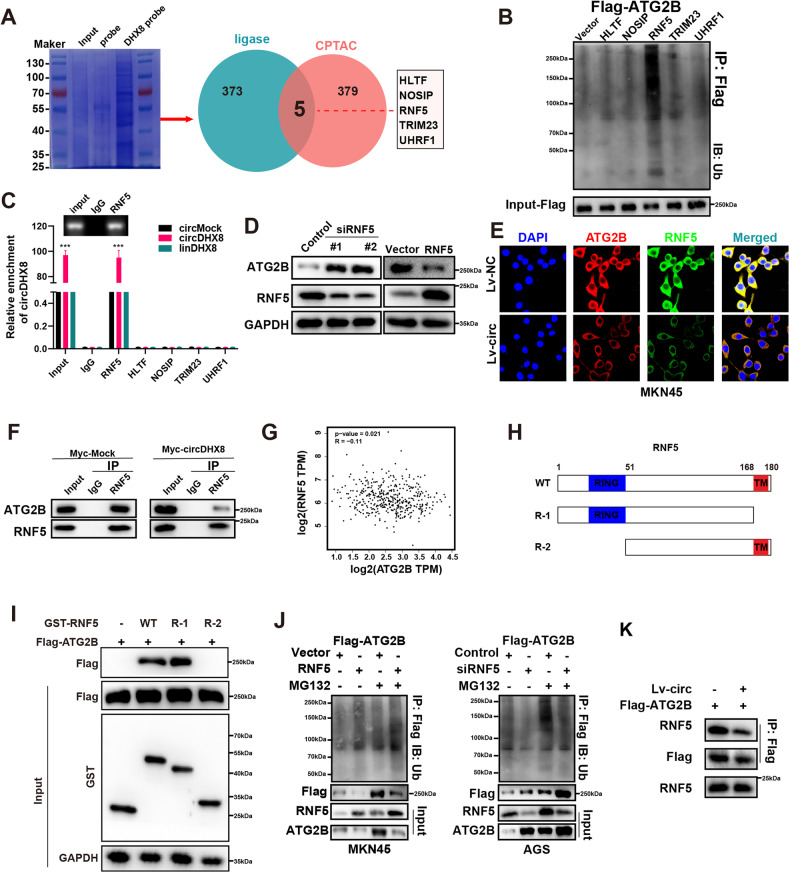
CircDHX8 inhibits the ATG2B‑RNF5 interaction. **A** Proteins associated with the ubiquitination process that may bind to circDHX8 were predicted. **B** Ubiquitination of ATG2B was analyzed by western blotting. **C** RNA immunoprecipitation assay indicated that RNF5 could bind with circDHX8. **D** Western blotting analysis of ATG2B protein levels after RNF5 overexpression and knockdown in gastric cancer (GC) cells. **E** Immunofluorescence staining assays indicating the colocalization of RNF5 (green) and ATG2B (red) in AGS and MKN45 cells, with nuclear staining using DAPI (blue). **F** Co-immunoprecipitation analysis of mutual interaction between RNF5 and ATG2B. **G** Analysis of correlation between ATG2B and RNF5 mRNA in GC from GEPIA database. *P* value derived from Pearson correlation analysis. **H** Schematic diagram indicating the domains of RNF5 truncations. **I** In vitro binding assay showing specific domains in which ATG2B binds RNF5. **J** The function of RNF5 in the ubiquitination of ATG2B was analyzed by western blotting and immunoprecipitation. **K** The influence of circDHX8 on the mutual interaction between RNF5 and ATG2B was analyzed by western blotting and immunoprecipitation. ***P < 0.001.

To elucidate how circDHX8 influences ATG2B ubiquitination in GC, we performed RNA pull-down assays using a biotin-labeled probe targeting the circDHX8 back-splicing site in GC cells. Cross-referencing of the proteins identified in the pull-down assay using the CPTAC database revealed that circDHX8 bound to five ubiquitin-related proteins that were differentially expressed in GC cells (Fig. 5A). Subsequent experiments established that RNF5 significantly enhanced ATG2B ubiquitination (Fig. 5B). RIP assays further confirmed RNF5’s binding to circDHX8, as opposed to linear DHX8 (Fig. 5C). Construction of RNF5 knockdown and overexpression plasmids demonstrated that RNF5 reduced ATG2B expression, with immunofluorescence and immunoprecipitation experiments indicating that circDHX8 could inhibit the interaction between RNF5 and ATG2B (Fig. 5D–F). However, analysis of RNF5 and ATG2B mRNA expression in GC from the GEPIA database showed no significant correlation (Fig. 5G). Therefore, we hypothesized that circDHX8 enhances ATG2B stability by preventing ATG2B binding to RNF5, thereby modulating its biological functions. In vitro binding assays revealed that the R-1 domain of GST-tagged RNF5 is critical for its interaction with ATG2B (Fig. 5H, I). Further experiments demonstrated that increased RNF5 expression increased ATG2B ubiquitination, whereas RNF5 knockdown had the opposite effect (Fig. 5J). Notably, circDHX8 upregulation also inhibited RNF5 binding with ATG2B (Fig. 5K). In conclusion, our results confirmed that circDHX8 stabilizes ATG2B and inhibits its ubiquitination by competitively binding to RNF5.

### Starvation could induce acetylation of ATG2B, which inhibits the binding of ATG2B to RNF5

**Fig. 6:**
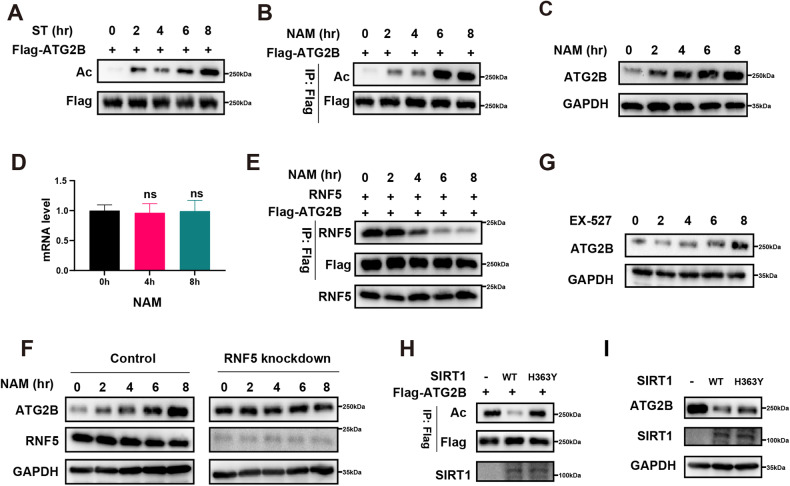
ATG2B-RNF5 interaction depends on ATG2B deacetylation. **A** Acetylation levels of ATG2B were analyzed by western blotting after starvation (ST) treatment. **B** Acetylation levels of ATG2B were analyzed by western blotting after NAM treatment. **C** Western blotting analysis of ATG2B protein levels after NAM treatment. **D** qPCR analysis of ATG2B after NAM treatment. **E** The influence of NAM on the RNF5 mutual combination of ATG2B was analyzed by western blotting and immunoprecipitation. **F** Western blotting analysis of ATG2B protein levels after NAM treatment and RNF5 knockdown. **G** Western blotting analysis of ATG2B protein levels after EX-527 treatment. **H** The influence of SIRT1 on ATG2B acetylation was analyzed by western blotting and immunoprecipitation. **I** The influence of SIRT1 on ATG2B protein levels was analyzed by western blotting.

Previous studies have revealed that acetylation could participate in autophagy regulation, and many autophagy-related proteins (ATGs) could undergo acetylation or deacetylation [26–30]. To investigate whether ATG2B undergoes acetylation or deacetylation during autophagy, we utilized a specific antibody against acetylated lysine to examine ATG2B acetylation in GC cells induced by starvation (ST). Unexpectedly, we found that cell starvation stimulated ATG2B acetylation (Fig. 6A). Treating GC cells with nicotinamide (NAM), a SIRT family deacetylase inhibitor, resulted in marked acetylation in NAM-treated cells, which intensified over time (Fig. 6B). Following extended processing times with NAM, the expression of ATG2B protein in GC cells was significantly increased (Fig. 6C). qPCR analysis indicated that NAM did not alter the ATG2B mRNA levels (Fig. 6D). Immunoprecipitation assays revealed that NAM treatment weakened the binding between ATG2B and RNF5 in GC cells and that RNF5 knockdown inhibited the effect of NAM on ATG2B protein expression (Fig. 6E, F). These results confirm that acetylation significantly impedes the binding of ATG2B to RNF5. To further confirm that ATG2B deacetylation was dependent on SIRT1, we treated GC cells with EX-527, a SIRT1 inhibitor (Fig. 6G). Additionally, we overexpressed both wild-type SIRT1 (SIRT1 WT) and a SIRT1 mutant (SIRT1-H363Y) and observed that the SIRT1-H363Y mutation did not significantly affect ATG2B acetylation or protein levels (Fig. 6H, I). Collectively, these findings suggest that ATG2B acetylation can be induced by cell starvation, and acetylation could inhibit the binding of ATG2B to RNF5.

### Effect of circDHX8, ATG2B, and RNF5 on autophagy, proliferation, and aggressiveness of GC cells

**Fig. 7:**
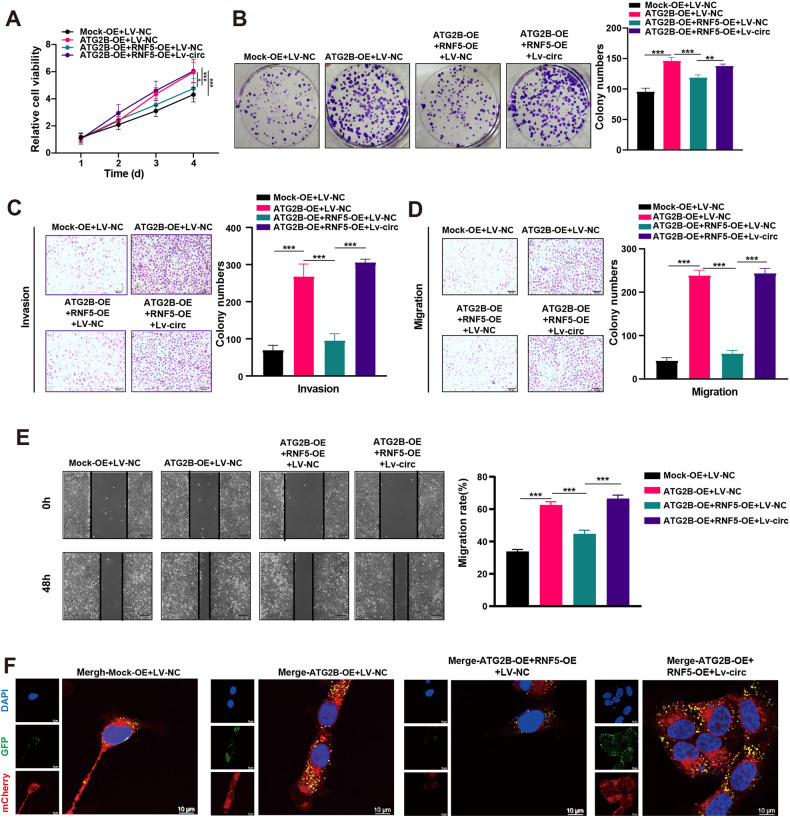
Effects of circDHX8, ATG2B, and RNF5 on the growth and aggressiveness of GC cells. **A** Cell counting kit-8 analysis of cell proliferation in MKN45 transfected with corresponding plasmids. **B** Colony-forming capacity of MKN45 transfected with corresponding plasmids. **C**, **D** Transwell assays for determining the migration and invasion abilities of MKN45 cells transfected with corresponding plasmids. **E** A wound healing assay was performed to evaluate the migration ability of MKN45 cells transfected with corresponding plasmids. **F** Immunofluorescence staining of autophagosomes and autolysosomes in MKN45 cells transfected with corresponding plasmids. **P < 0.01 and ***P < 0.001.

We investigated the functional relationships between circDHX8, ATG2B, and RNF5 in AGS and MKN45. Cell counting kit-8 and colony formation assays showed that RNF5 overexpression mitigated the proliferation promotion effect induced by ATG2B overexpression in GC cells, and this effect was restored by circDHX8 upregulation (Fig. 7A, B). In contrast, the inhibitory effect on proliferation generated by ATG2B knockdown could be ameliorated by depleting RNF5. circDHX8 knockdown counteracted the effects of RNF5 (Fig. S4A, B). Transwell and wound healing assays also showed that ATG2B overexpression promoted the invasion and migration of GC cells, and the upregulation of RNF5 reversed this effect. circDHX8 overexpression counteracted the effect of RNF5 (Fig. 7C–E), and knockdown experiments resulted in the opposite results (Fig. S4C–E). We previously confirmed that circDHX8 promotes autophagy in GC cells through ATG2B and that this function can be restored by the upregulation of RNF5 (Fig. 7F). In conclusion, circDHX8 competitively binds to RNF5, preventing the interaction between ATG2B and RNF5 to promote ATG2B protein stability in GC cells, thus promoting autophagy and tumor progression in GC.

### CircDHX8 and ATG2B overexpression correlates with unfavorable pathological characteristics in GC

**Fig. 8:**
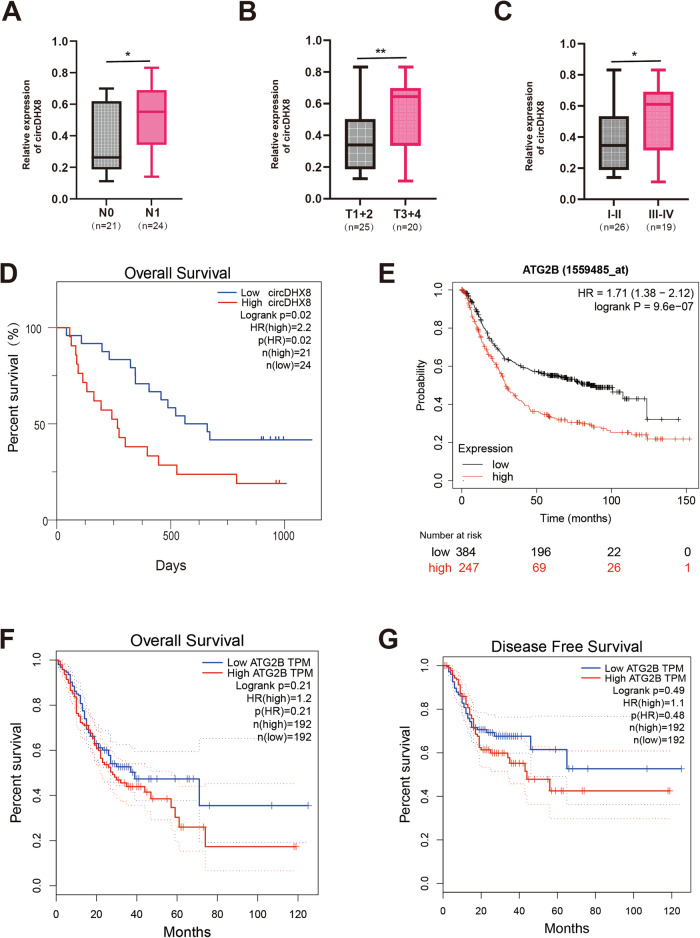
Higher expression of circDHX8 and ATG2B correlates with unfavorable pathological characteristics in gastric cancer (GC). **A**–**C** qPCR analysis showed that circDHX8 mRNA expression levels correlated with clinicopathological characteristics. *P* value derived from the χ^2^ test. **D** Log-rank test for survival comparison between high circDHX8 (*n* = 21) and low circDHX8 (*n* = 24) in GC. **E** Survival curves of ATG2B expression in patients with GC from the Kaplan–Meier plotter. **F**–**G** Analysis of overall survival curves and disease-free survival in GC based on TCGA data from GEPIA.

Finally, through the analysis of the correlation of circDHX8 expression and clinical pathological information, we found that patients with advanced GC had significantly higher circDHX8 expression and a worse prognosis (Fig. 8A–D). Additionally, the Kaplan–Meier curves of overall survival using the Kaplan–Meier plotter (http://kmplot.com/analysis/) showed that higher ATG2B expression is associated with worse prognosis (Fig. 8E). Although there was no statistically significant difference, TCGA data from GEPIA (http://gepia.cancer-pku.cn/detail.php) also demonstrated that high ATG2B expression correlated with poor overall survival and disease-free survival in patients with GC (Fig. 8F, G). These results indicated that circDHX8 and ATG2B have the potential to predict GC prognosis.

## Discussion

Approximately 10 years ago, the clinical significance of circRNAs was first uncovered, suggesting their potential involvement in human diseases [31, 32]. Advances in RNA sequencing technology have significantly enhanced our understanding of circRNAs. Recent investigations have revealed that a substantial number of circRNAs are abnormally expressed in nearly all cancer types and are pivotal for cancer initiation and progression [14]. For instance, hsa_circ_0006401 improves cell proliferation and metastasis by enhancing the stability of the host gene, col6a3 mRNA, in colorectal cancer [33]. Furthermore, hsa_circ_0110389/miR-127-5p/SORT1 promotes GC progression [34]. circBACH2 enhanced proliferation, invasion, and migration in triple-negative breast cancer [35]. circGSK3B also promotes RORA expression to suppress GC progression by preventing EZH2 transinhibition [24]. However, the specific functions of circDHX8 in GC and its underlying mechanisms have not been elucidated. Our research confirmed that circDHX8 was highly expressed in GC samples and cell lines. Subsequently, our in vitro and in vivo functional experiments confirmed that circDHX8 enhanced the malignancy of gastric cancer cells.

Autophagy is generally considered a protective mechanism against cellular damage [36]. Recent studies have revealed aberrant autophagy in cancer [37–39]. Considering the multifaceted and varied functions of autophagy in cancer, its role in cancer treatment remains unclear. Some autophagy-related circRNAs have been identified to play roles in GC progression. For example, circCUCL2 induces autophagy and regulates the malignant transformation of GC through miR-142-3p/ROCK2 [5]. Moreover, circRELL1 could promote GC progression by activating autophagy as a miR-637 sponge [40]. We observed that circDHX8 may exert its biological function in GC cells by promoting autophagy. These previous findings combined with our current results suggest the importance of the role circRNAs on autophagy and promoting cancer progression.

Most autophagy-related circRNAs function as miRNA sponges [14]. However, circRNAs can also regulate gene expression by interacting with specific RNAs [41] and binding to RNA-binding proteins such as protein scaffolds or antagonists [42, 43]. A few circRNAs can even undergo direct translation into proteins [44, 45]. Our research uncovered a novel mechanism by which autophagy-related circDHX8 binds to the ubiquitinase RNF5 and inhibits its interaction with its target ATG2B. ATG2B is a newly discovered protein involved in autophagy progression, and ATG2B is essential for autophagosome formation [46]. Thus, we assume that circDHX8 promotes autophagy in GC cells by deubiquitinating and stabilizing ATG2B, thereby exerting its biological function in GC cells.

Humans have 16 known autophagy-related genes (ATG). Abnormal mutations in ATG2B may influence cancer development through dysregulated autophagy [47–49]. However, the specific regulatory mechanisms involving circRNAs and ATG2B remain unclear. RIP and RNA pull-down assays suggested that circDHX8 did not directly bind to ATG2B but inhibited ATG2B ubiquitination and promoted its stabilization. Furthermore, by cross-referencing the proteins identified in the probe pull-down experiment and the CPTAC database, we observed that circDHX8 interacted with RNF5, which significantly enhanced ATG2B ubiquitination. Immunofluorescence and co-immunoprecipitation assays confirmed the interaction between ATG2B and RNF5. Based on these findings, we hypothesized that circDHX8 acts as an antagonist by inhibiting the binding of RNF5 to ATG2B. Subsequent IP assays provided evidence supporting this hypothesis.

Moreover, none of the cellular processes were determined by unilateral factors. We uncovered a key mechanism for the acetylation of autophagy-related genes, at least in starved cells. Accumulating evidence demonstrates that acetylation of autophagy-associated proteins affects protein stability [50–52]. In our study, the binding of ATG2B to the ubiquitination-related protein RNF5 was not only influenced by circDHX8 but was also regulated by its own acetylation level. Under normal circumstances, the ubiquitination degradation of ATG2B can be regulated by its own acetylation level; however, the acetylation level of ATG2B is regulated by the cellular trophic status and intracellular deacetylase SIRT1. Perhaps this delicate balance maintains normal body function; notwithstanding, circDHX8 overexpression disrupts this balance and promotes GC progression.

In conclusion, we investigated the functional relationships between circDHX8, ATG2B, and RNF5 in GC cells. RNF5 overexpression mitigated the proliferation and migration promotion effects induced by ATG2B overexpression in GC cells, and this effect was restored by circDHX8 upregulation. The knockdown experiments led to the opposite results. Thus, circDHX8 promotes autophagy and tumor progression in GC by binding to the ubiquitinase RNF5 to prevent the interaction between ATG2B and RNF5 to stabilize ATG2B. According to the clinical data from our center, we found that patients with advanced GC had a significantly higher circDHX8 expression and a worse prognosis. Taken together, our research indicates that circDHX8 and ATG2B have the potential value to predict GC prognosis and may act as treatment targets. However, there remain some limitations in this research. The specific mechanism by which circDHX8 can competitively bind to RNF5 remains unclear. The molecular mechanism by which starvation stress activates ATG2B acetylation in gastric cancer cells needs further clarification, and whether circDHX8 affects the acetylation level of ATG2B remains to be demonstrated. Answering these questions in our follow-up study will contribute to the further improvement of targeted therapy for gastric cancer.

### Supplementary information

Supplementary_Figure

Full and uncropped western blots

## Data Availability

The data generated in this study are available upon request from the corresponding author.
