# Supplementary material for: Human gastric cancer progression and stabilization of ATG2B through RNF5 binding facilitated by autophagy-associated CircDHX8
Source: Cell Death Dis. 2024 Jun 12;15(6):410. doi: 10.1038/s41419-024-06782-8 (PMC11169566; doi:10.1038/s41419-024-06782-8)
Supplement: Supplementary file 1 — Supplementary_Figure [file 41419_2024_6782_MOESM1_ESM.pdf]

Figure S1

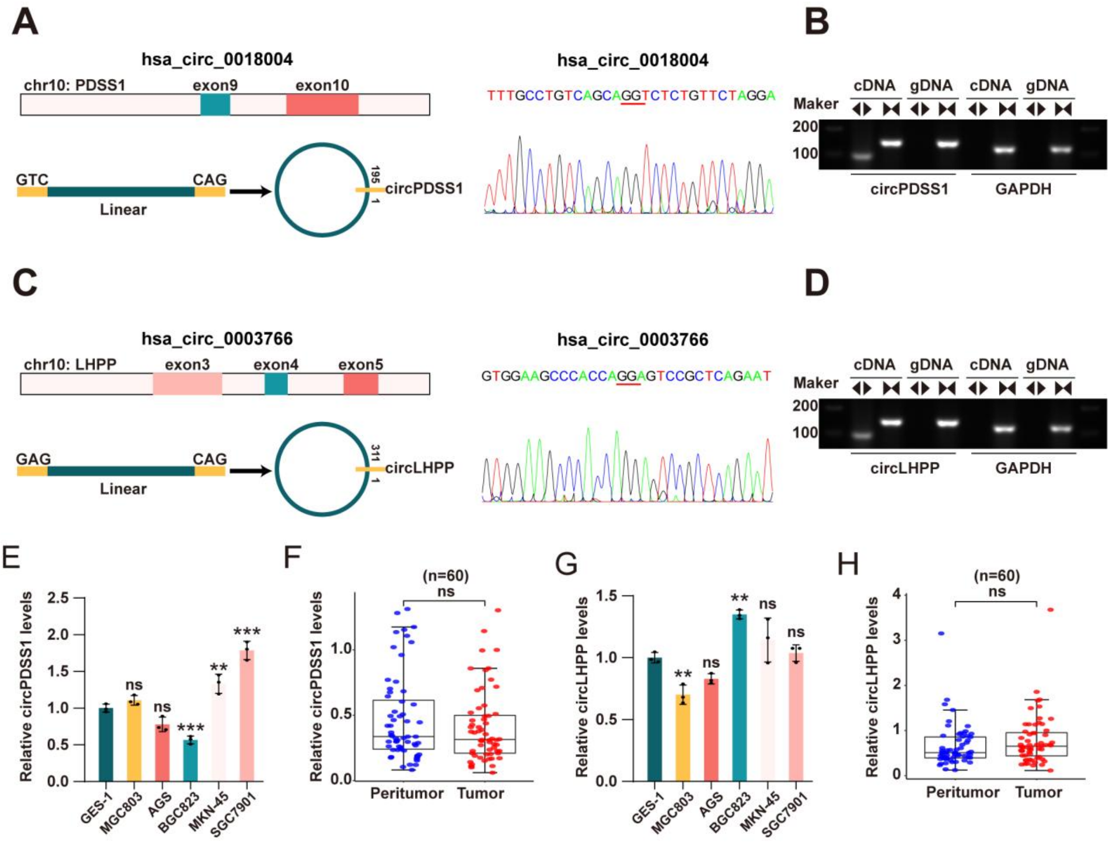

Figure S1 . A–D Sanger sequencing and DNA electrophoresis results with the PCR products of hsa_circ_0018004 and hsa_circ_0003766. **E** qPCR analysis of circPDSS1 in GC cells and GES-1 cells. **F** qPCR analysis of circPDSS1 in GC and peritumor tissues (n = 60). **G** qPCR analysis of circLHPP in GC cells and GES-1 cells. **H** qPCR analysis of circLHPP in GC and peritumor tissues (n = 60).

GC, gastric cancer.

Figure S2

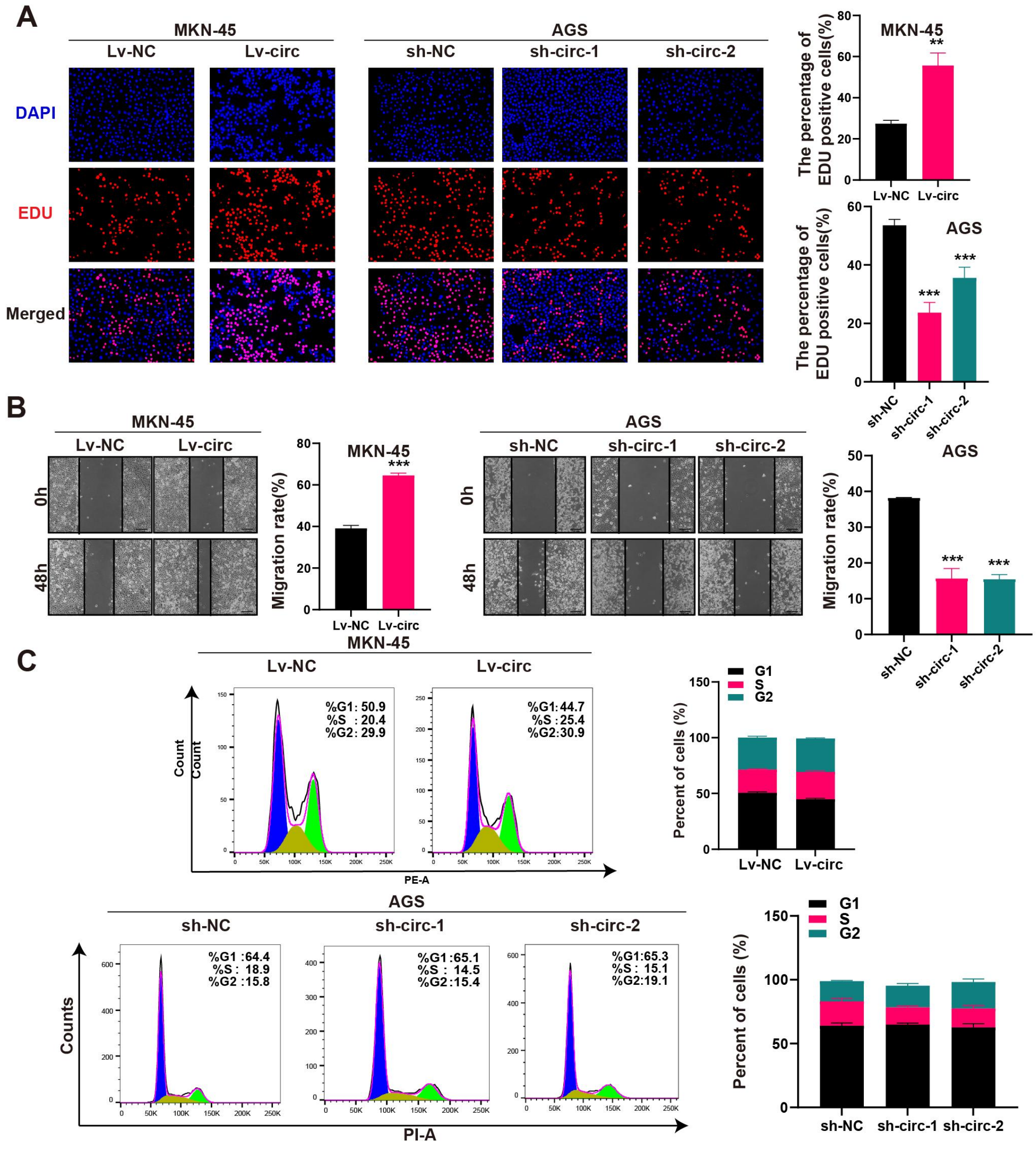

Figure S2 . A EdU assay of cell proliferation in MKN45 and AGS cells after circDHX8 overexpression or knockdown. **B** Wound healing assay of cell migration in MKN45 and AGS cells after circDHX8 overexpression or knockdown. **C** Flow cytometry was performed to assess the impact of circGSK3B on the cell cycle of GC cells.

GC, gastric cancer

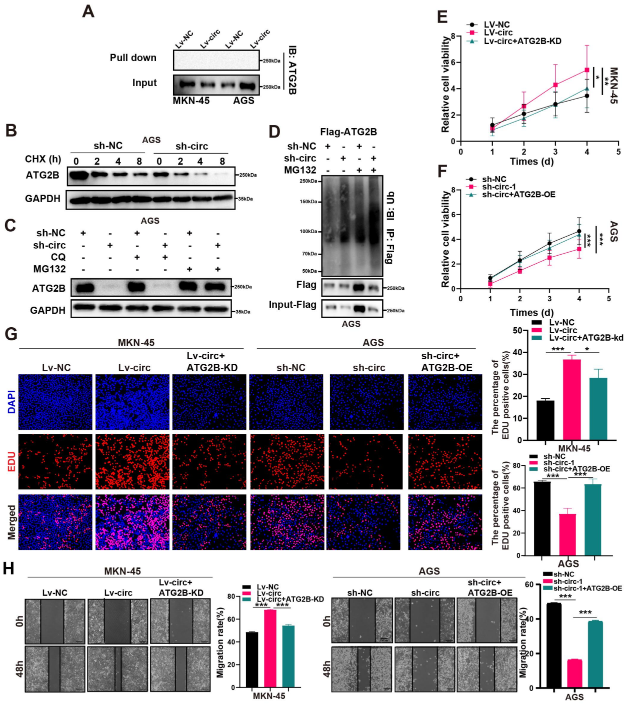

Figure S3

Figure S3 . A RNA pull-down analysis of the relative interplay between circDHX8 and ATG2B. **B** Western blotting analysis of the protein stability of ATG2B after circDHX8 knockdown.**C** The effect of circDHX8 and CQ on ATG2B protein levels was analyzed by western blotting.**D** Western blotting assay showed that circDHX8 knockdown could promote the ubiquitination of ATG2B. **E-G** CCK-8 assays and EdU assays of cell proliferation. **H** Wound healing assay of cell migration. CCK-8: cell counting kit-8; CQ, chloroquine

Figure S4

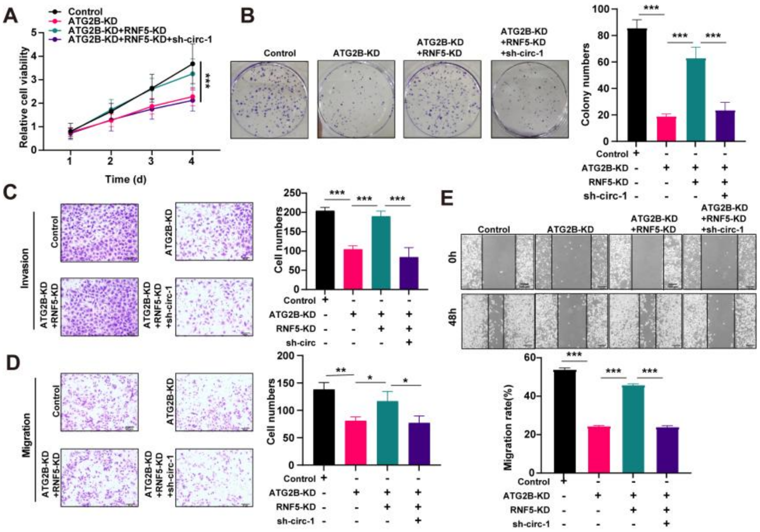

Figure S4. A–B CCK8 and colony formation assays were used to analyze cell proliferation. **C–E** Transwell assays and wound healing assays were used to analyze cell migration and invasion in gastric cancer cells.
