# Supplementary figures and images for: Human gastric cancer progression and stabilization of ATG2B through RNF5 binding facilitated by autophagy-associated CircDHX8
Source: Cell Death Dis. 2024 Jun 12;15(6):410. doi: 10.1038/s41419-024-06782-8 (PMC11169566; doi:10.1038/s41419-024-06782-8)

**FIG 3**

**B**

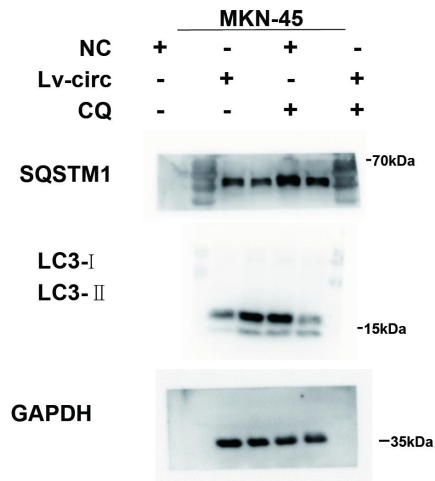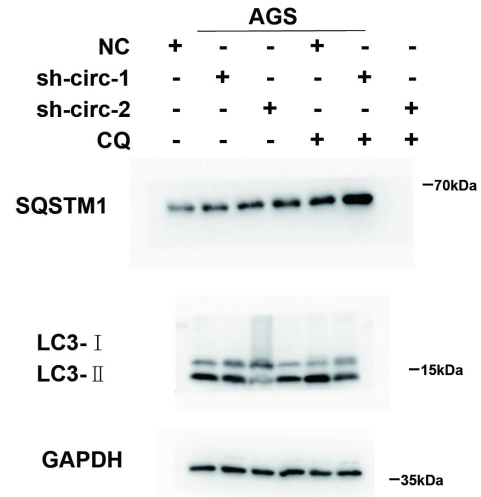

**C**

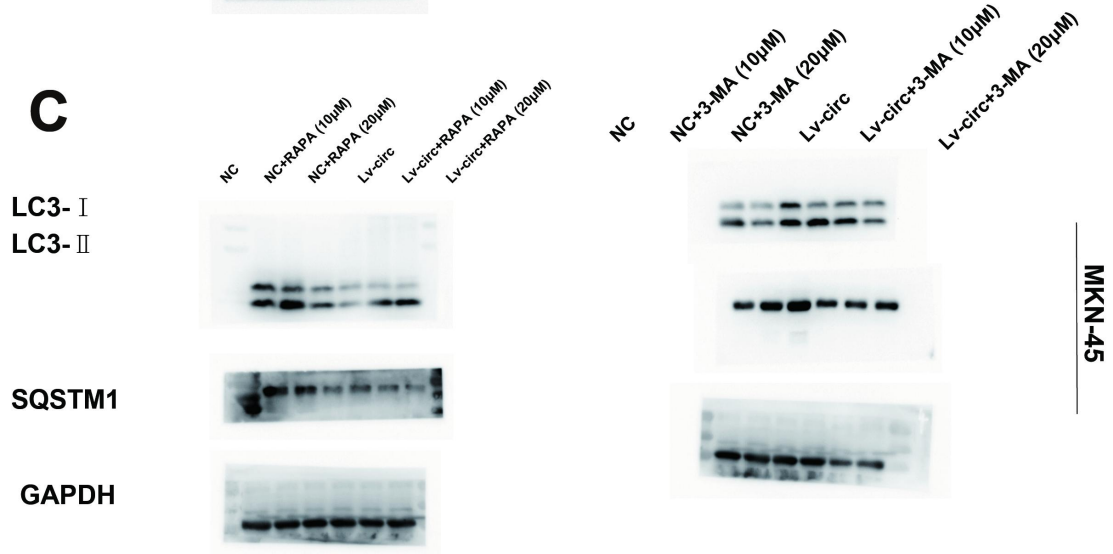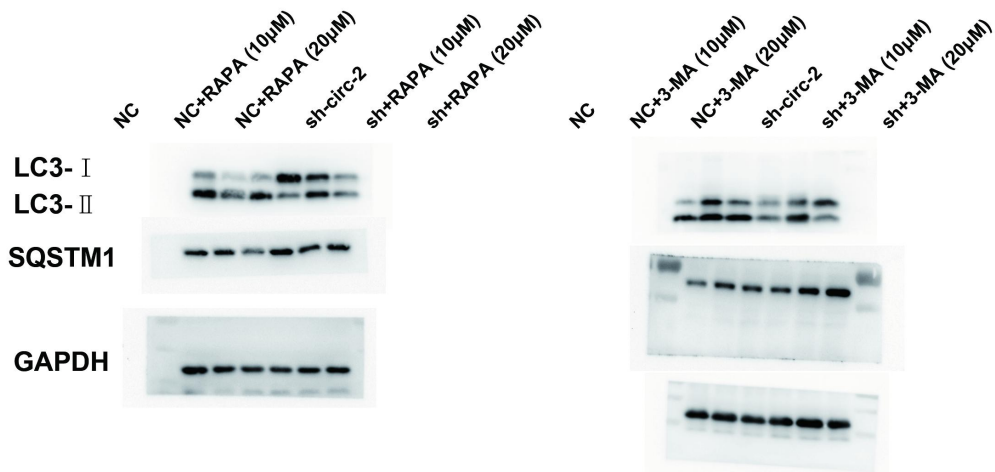

**FIG 4**

**E**

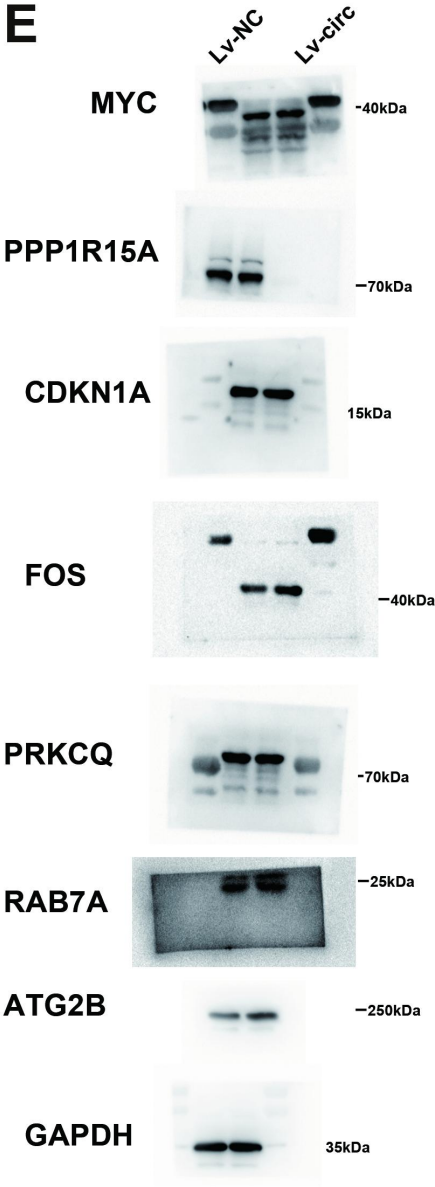

**F**

CHX (h)

ATG2B

GAPDH

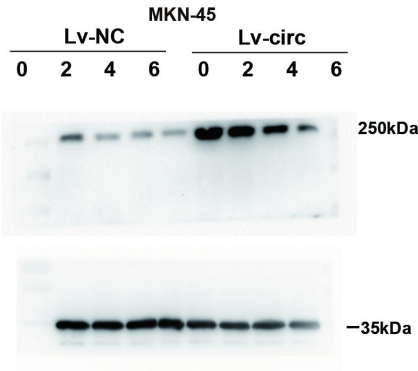

**G**

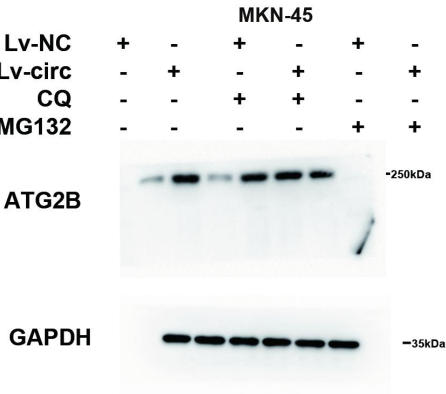

**H**

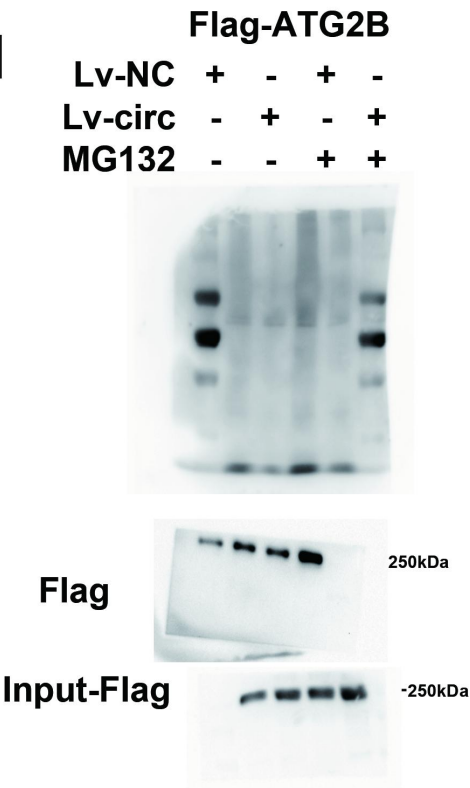

**FIG 5**

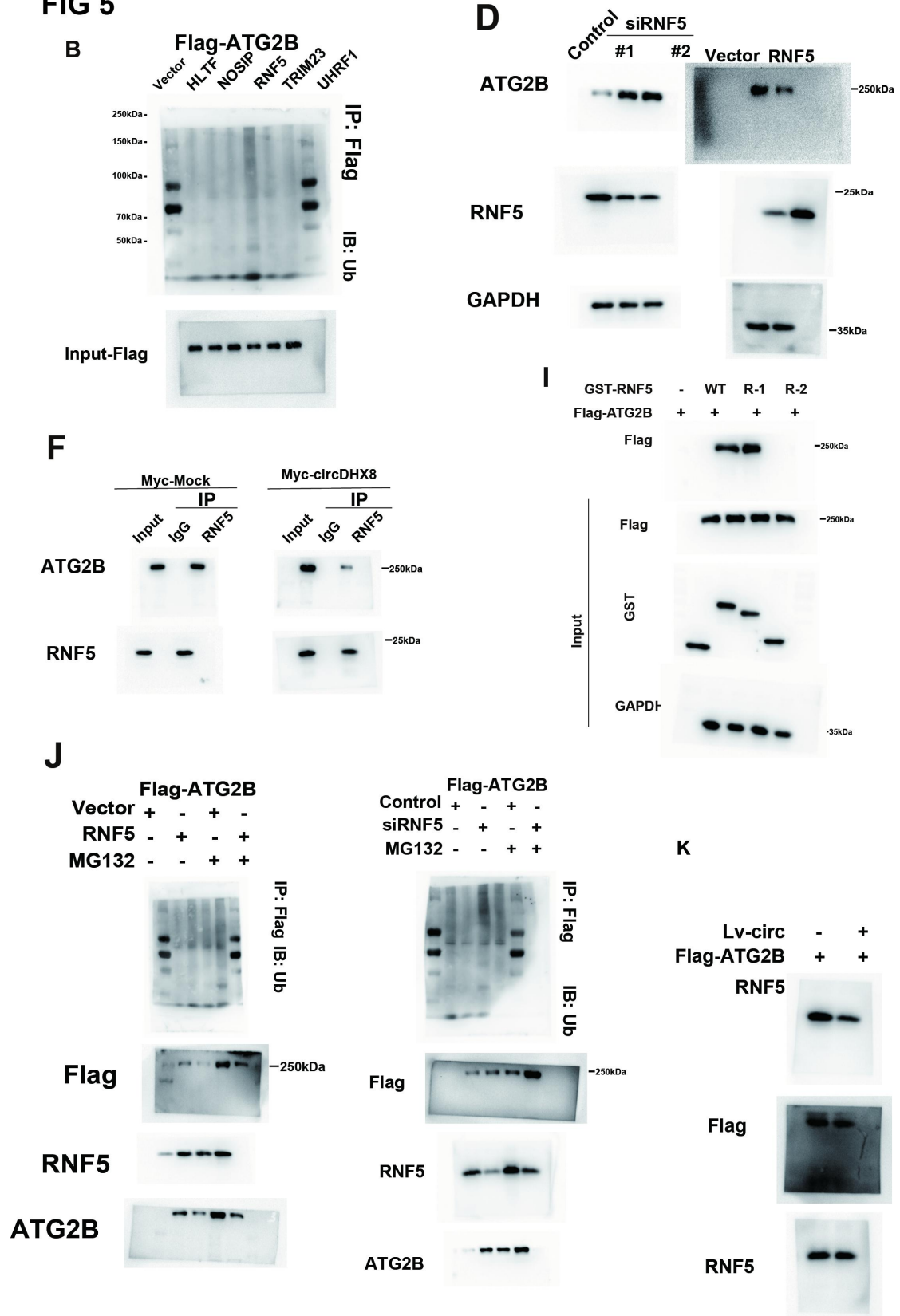

**FIG 7**

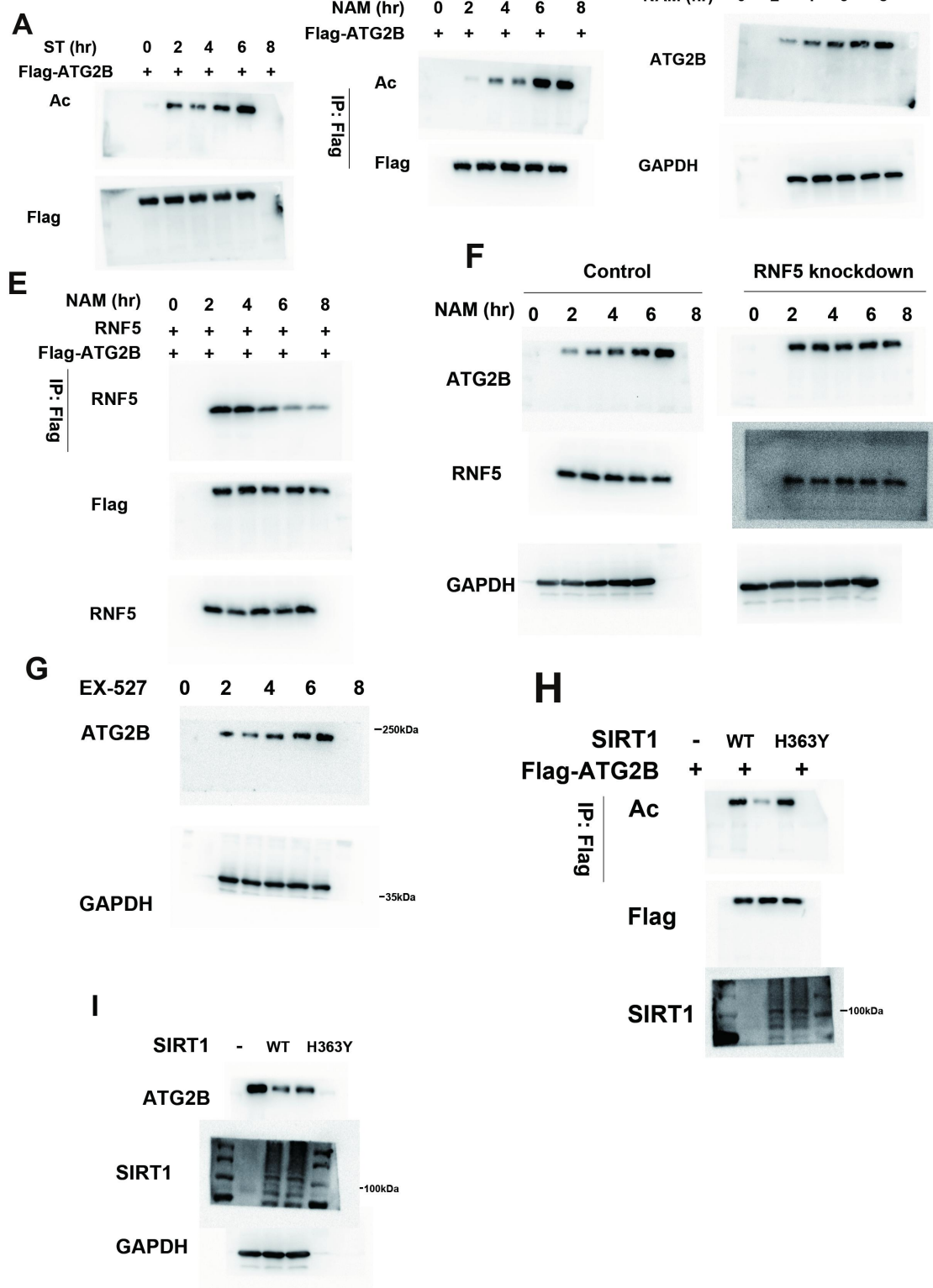

FIG S3

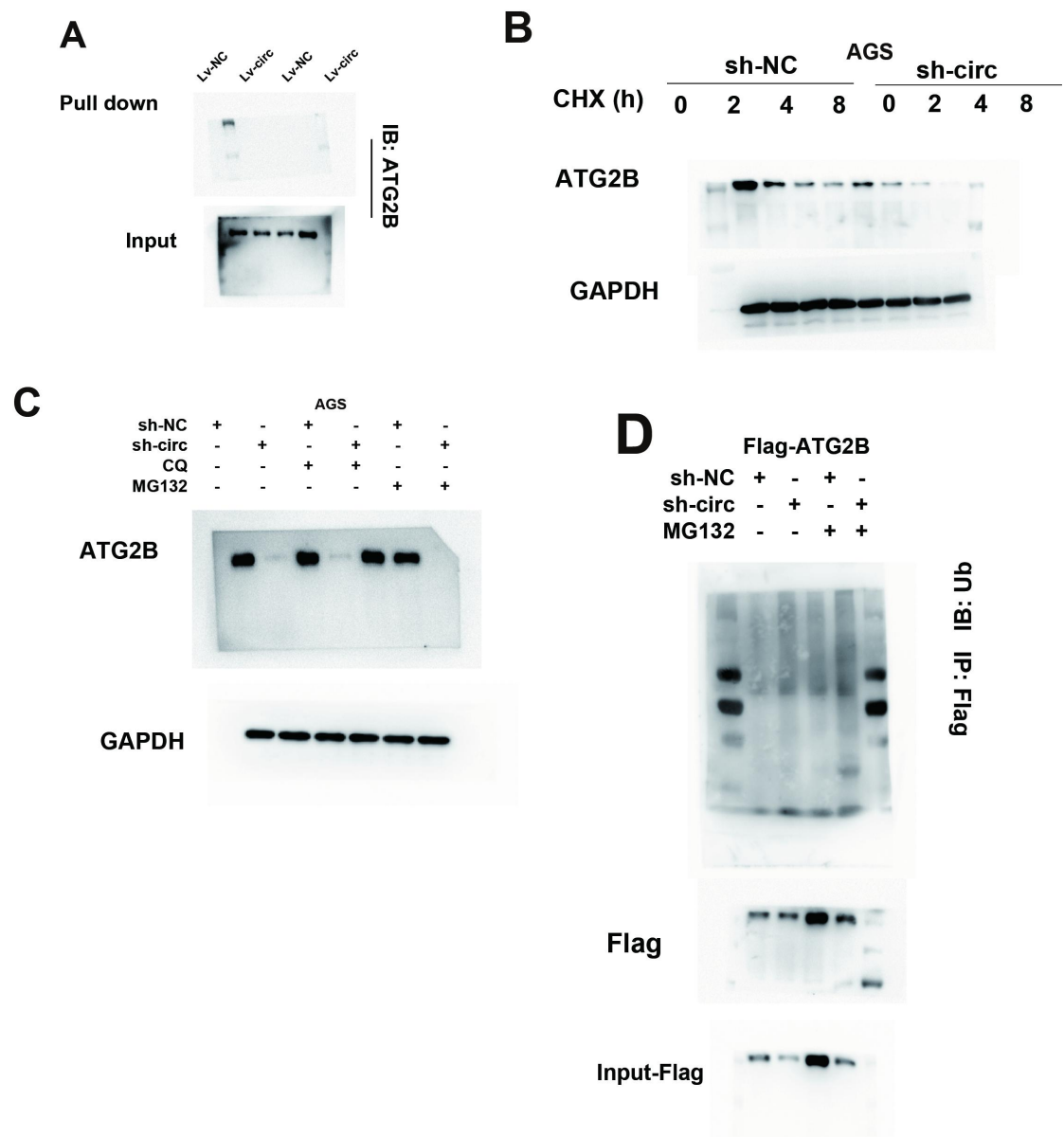

Supplement: Supplementary file 2 — Full and uncropped western blots [file 41419_2024_6782_MOESM2_ESM.pdf]
